# Multimodal Surgical Management of Stage 1a/1b PCFD (Stage II AAFD): Early Outcomes of a Standardized Four-in-One Procedure Protocol

**DOI:** 10.3390/diagnostics16081124

**Published:** 2026-04-09

**Authors:** Yu Ting Chen, Cing Syue Lin, Shou En Cheng, Shang Ming Lin, Tsung Yu Lan

**Affiliations:** 1 Medical Education Department, Far Eastern Memorial Hospital, No. 21, Sec. 2, Nanya S. Rd., New Taipei City 220, Taiwan; arielchen825@gmail.com; 2 Department of Orthopedic Surgery, Far Eastern Memorial Hospital, No. 21, Sec. 2, Nanya S. Rd., New Taipei City 220, Taiwan; salantfg@gmail.com (C.S.L.); samuel830125@gmail.com (S.E.C.); 3 Department of Materials and Textiles, Asia Eastern University of Science and Technology, No. 58, Sec. 2, Sihchuan Rd., New Taipei City 220, Taiwan; fc013@mail.aeust.edu.tw

**Keywords:** progressive collapsing foot deformity, standardized reconstruction, peritalar instability, internal brace, subtalar arthroereisis, joint-preserving surgery

## Abstract

**Background/Objectives:** Progressive collapsing foot deformity (PCFD) is driven by multiplanar peritalar instability. This study evaluated the clinical and radiographic outcomes of a standardized four-component reconstruction protocol designed to facilitate immediate postoperative weight-bearing in Stage 1a/1b PCFD. **Methods:** This single-center retrospective study included 20 patients treated between 2015 and 2023 with medializing calcaneal osteotomy, spring ligament repair, flexor digitorum longus (FDL) tendon transfer with internal brace augmentation, and subtalar arthroereisis. Clinical (VAS, AOFAS) and radiographic parameters (anteroposterior and lateral Meary angles, calcaneal pitch, and talonavicular coverage angle) were assessed longitudinally, with subgroup analysis comparing implant removal versus retention. **Results:** The protocol yielded significant overall improvements. Mean VAS decreased by 4.37 points (*p* < 0.001), and final AOFAS reached 84.7 ± 7.6 at the final follow-up. Although subtalar arthroereisis was removed in 45% of patients due to symptomatic irritation, subgroup analysis revealed no significant loss of radiographic correction (*p* > 0.05). Notably, a significant interaction effect was observed for VAS scores (*p* = 0.002) and AOFAS scores (*p* = 0.041), with the removal group demonstrating a pronounced functional recovery trajectory following explantation. No major complications occurred. **Conclusions:** A standardized four-in-one reconstruction provides reliable multiplanar correction in Stage 1a/1b PCFD. The maintenance of structural alignment despite a high implant removal rate supports the role of arthroereisis as a temporary but valuable adjunct for early mobilization. This strategy offers a reproducible framework for joint-preserving PCFD management.

## 1. Introduction

Progressive collapsing foot deformity (PCFD), traditionally known as adult-acquired flatfoot deformity (AAFD), is a complex, three-dimensional pathology characterized by loss of the medial longitudinal arch, hindfoot valgus, and forefoot abduction [1,2,3]. While historically viewed through a tendon-centric lens, modern understanding emphasizes a multifactorial failure of both dynamic stabilizers, such as the posterior tibial tendon, and critical static restraints; namely, the spring ligament complex [1]. Clinically, symptomatic PCFD is associated with pain, fatigue, gait disturbance, and measurable functional limitations that can substantially impair the activities of daily living [3,4]. In the early stages of the disease (PCFD Stage 1a and 1b; or AAFD stage II), the deformity remains flexible, offering an optimal window for joint-preserving surgical interventions.

The pathogenesis of PCFD is multifactorial and involves the failure of dynamic stabilizers—most notably, the posterior tibial tendon—and static medial restraints, including the spring ligament complex and its continuity with the deltoid ligament [3,4,5,6]. Increasing biomechanical and imaging evidence suggests that insufficiency of these medial ligamentous structures plays a central role in permitting progressive peritalar subluxation, valgus collapse, and medial column instability [5,6,7]. Once static restraint is compromised, compensatory overload of remaining structures accelerates deformity progression, even in the presence of a functionally intact tendon [7,8]. Imaging plays a central role in diagnosis, phenotyping, and longitudinal assessment, with weight-bearing radiographs serving as the minimum standard. Weight-bearing CT is recommended for the surgical planning and assessment of peritalar subluxation [3,9,10]. Consequently, early-stage PCFD represents a critical window during which comprehensive joint-preserving reconstruction may halt disease progression and restore physiological alignment.

Stage II AAFD, broadly corresponding to Stage 1 PCFD (stages 1a and 1b), is defined as a flexible deformity without fixed hindfoot valgus or advanced subtalar arthritis. This stage is considered optimal for reconstructive interventions [1,4,11]. A wide range of operative techniques has been described, including medializing calcaneal osteotomy (MCO), flexor digitorum longus (FDL) tendon transfer, spring ligament repair or reconstruction, lateral column procedures, and subtalar arthroereisis [4,6,12,13]. While each technique addresses a specific component of the deformity, no single procedure reliably corrects the full spectrum of hindfoot valgus, medial column collapse, and ligamentous insufficiency that characterizes early-stage PCFD [3,6,11]. Specifically, MCO utilizes a medial slide of the calcaneal tuberosity to realign the Achilles vector, while FDL transfer and spring ligament procedures focus on augmenting dynamic tendon function and stabilizing the medial longitudinal arch.

Practice variation remains substantial in operative selection and sequencing, reflecting differences in surgeon preference and deformity interpretation. Registry-based data suggest that procedure choice often varies according to severity and patient factors [14,15]. Increasing attention has shifted toward multimodal, protocol-driven reconstructions that concurrently address hindfoot valgus, dynamic tendon insufficiency, and static medial ligament restraints [6,11,12]. In particular, suture-tape (“internal brace”) augmentation has been introduced to reinforce spring and/or deltoid-spring repair during early healing and to resist elongation under physiologic load [12,16,17]. This synthetic reinforcement addresses recurrent instability, a known risk of isolated soft-tissue plication. Subtalar arthroereisis has also been proposed as an adjunctive method to temporarily limit excessive subtalar eversion while preserving motion; however, adult outcomes and implant tolerance remain controversial due to potential complications such as sinus tarsi pain and the subsequent need for implant removal [18,19,20].

Given the growing interest in comprehensive reconstruction and the persistent heterogeneity of operative algorithms, the outcomes of standardized multimodal protocols performed in a consistent sequence remain incompletely defined in early-stage PCFD [11,15,18]. Accordingly, the present study evaluated the early clinical and radiographic outcomes of a standardized four-in-one reconstruction protocol (MCO, FDL transfer, spring ligament repair augmented with internal bracing, and subtalar arthroereisis) for stages 1a and 1b PCFD [1,12,18]. This protocol systematically integrates bony realignment via a MCO slide with soft-tissue stabilization and temporary sinus tarsi implantation to address all facets of the deformity. We hypothesized that this combined approach would provide reliable deformity correction and meaningful functional improvement, while supporting earlier postoperative mobilization without compromising alignment [11,12,21].

## 2. Materials and Methods

### 2.1. Study Design and Clinical Evaluation

This is a comprehensive overview of a single-center retrospective cohort study evaluating standardized surgical reconstruction for stage 1a or 1b PCFD. This study included patients treated between 2015 and 2023 at the Far Eastern Memorial Hospital’s Department of Orthopedic Surgery, emphasizing a uniform approach to ensure reproducibility and minimize variability in outcomes.

Beginning with ethical considerations, institutional review board (IRB) approval was explicitly stated, and a waiver of informed consent due to the retrospective design was noted, which is a standard practice in such studies to facilitate data utilization while protecting patient privacy, as seen in similar orthopedic research. Patient selection criteria were clearly delineated, focusing on adults aged 50–80 years with early-stage PCFD confirmed through clinical examination (e.g., hindfoot valgus and inability to perform single-leg heel rise) and radiographic evidence (e.g., increased talonavicular coverage angle and reduced calcaneal pitch). Stratification into stages 1a and 1b addresses subtle differences in deformity progression, such as varying degrees of medial column instability or forefoot abduction, allowing for subgroup analyses that enhance the study’s analytical depth. The exclusion of advanced stages (III–IV) and confounding comorbidities ensured a homogeneous cohort, reduced bias, and improved internal validity.

**Figure 1:**
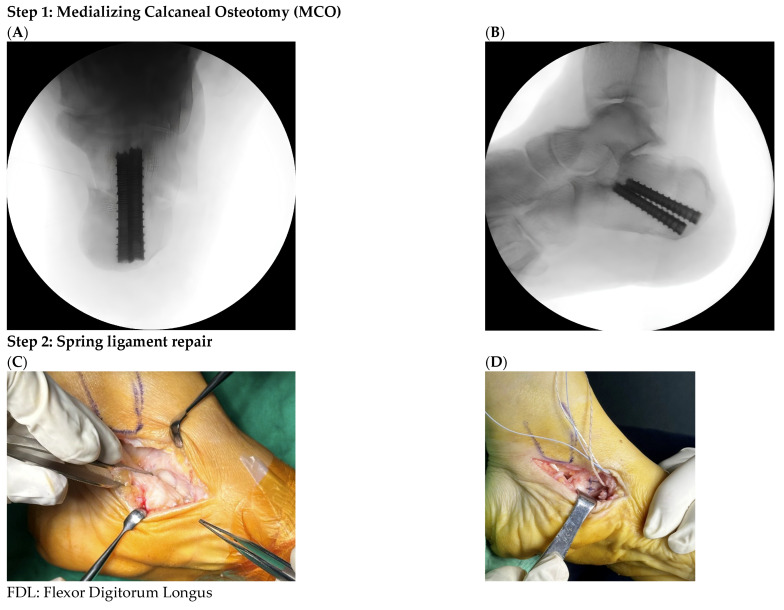

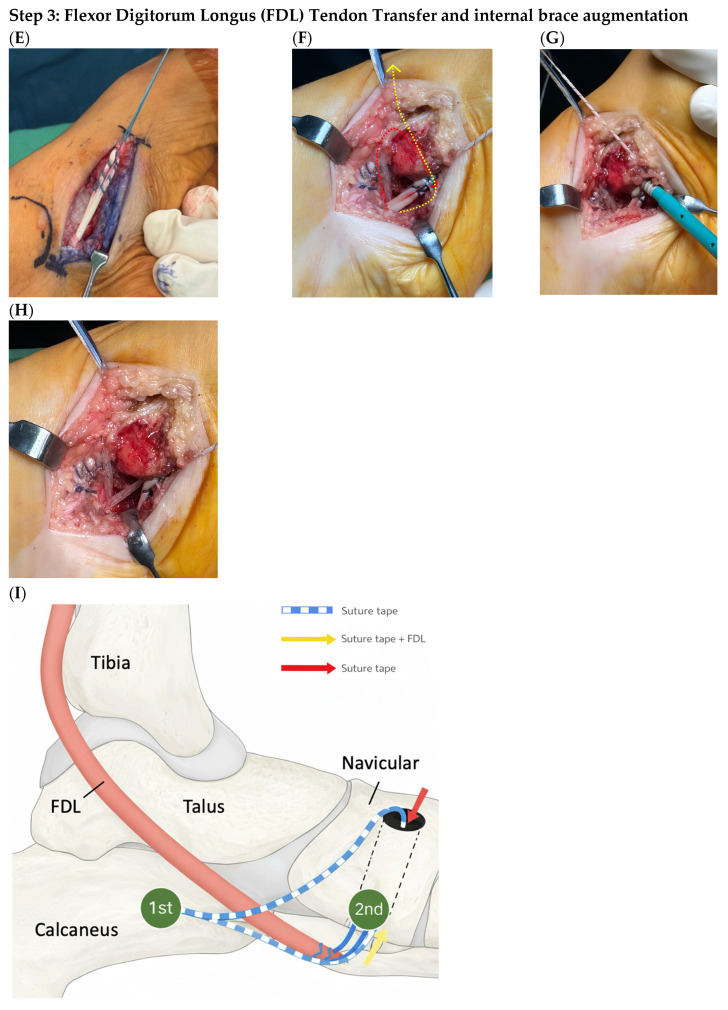

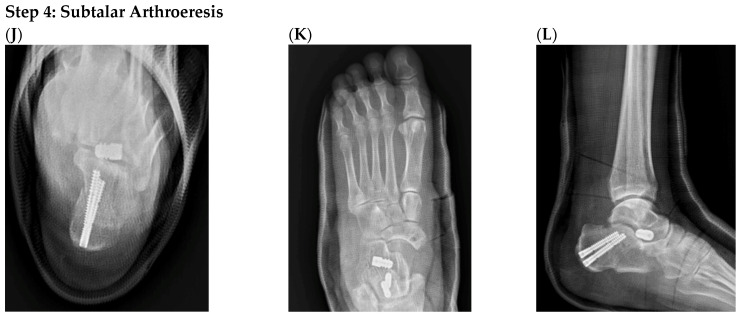
Stepwise illustration of the standardized four-in-one reconstruction protocol. Step 1: Medializing calcaneal osteotomy (MCO). Intraoperative fluoroscopic images in (**A**) axial and (**B**) lateral views confirming 8–12 mm medial translation of the calcaneal tuberosity. Fixation was achieved with two 7.0 mm headless compression cannulated screws to ensure hindfoot realignment and hardware stability. Step 2: Spring ligament repair. (**C**) Identify the rupture spring ligament (**D**) Spring ligament repair Step 3: Flexor Digitorum Longus (FDL) Tendon Transfer and internal brace augmentation. (**E**) Preparation of the flexor digitorum longus (FDL) tendon graft using the Krackow technique. (**F**) Placement of the first 4.75 mm suture anchor into the calcaneus, positioned inferior to the sustentaculum tali. Intraoperative view of the suture-tendon passage through the navicular bone tunnel; red dotted lines indicate the suture tape, and yellow dotted lines indicate the other suture tape and FDL. (**G**) The entire construct is tensioned and secured with a second 4.75 mm suture anchor inserted into the navicular bone tunnel from a plantar-to-dorsal direction. (**H**) Final intraoperative view of the tensioned triangular construct of suture tape and FDL tendon graft. (**I**) Schematic illustration showing the triangular framework formed by the suture tape and FDL tendon graft, providing both immediate static stabilization and dynamic medial column support. Step 4: Final stabilization with subtalar arthroereisis. Intraoperative fluoroscopic views in (**J**) axial, (**K**) anteroposterior, and (**L**) lateral views demonstrating the insertion of a sinus tarsi implant.

All procedures were performed by a single fellowship-trained foot and ankle surgeon, using a standardized four-in-one reconstruction protocol. The surgical steps were performed in a predefined sequence to address the multiplanar components of early-stage progressively collapsing foot deformities (Figure 1).

Step 1: Medializing Calcaneal Osteotomy (MCO). The procedure commenced with a MCO was performed using a lateral approach. Under fluoroscopic guidance, the calcaneal tuberosity was translated medially by approximately 8–12 mm to correct the hindfoot valgus and realign the pulling vector of the Achilles tendon from an evertor to an inversional force. Fixation was achieved using two 7.0 mm headless compression cannulated screw (Aplus, Taiwan, New Taipei City), and intraoperative fluoroscopy confirmed appropriate alignment and hardware position.

Step 2: Spring ligament repair. Subsequently, attention was directed toward the medial aspect of the foot. The spring ligament was initially addressed with direct repair using 1.0 Vicryl absorbable sutures.

Step 3: FDL Tendon Transfer and Internal Brace Augmentation. The posterior tibial tendon (PTT) was identified and inspected, followed by the isolation of the FDL tendon distal to the knot of Henry. The FDL tendon was harvested and prepared with No. 5 Ticron sutures (Medtronic, Minneapolis, MN, USA) using the Krackow sutures technique. A 4.75 mm suture anchor (SwiveLock; Arthrex, Naples, FL, USA) loaded with two bands of high-strength suture tape was inserted into the calcaneus, positioned inferior to the sustentaculum tali to provide a robust anatomical footprint for reconstruction. A bone tunnel was created at the navicular tuberosity using a drill. A wire passer was subsequently used to facilitate suture passage: one band of suture tape was passed through the tunnel in a superior-to-inferior direction, while the other band of suture tape together with the prepared FDL tendon was passed through the tunnel in an inferior-to-superior direction. The entire tape–FDL construct was tensioned and secured with a second 4.75 mm suture anchor into the navicular bone tunnel. This configuration provides immediate static and dynamic stabilization of the medial longitudinal arch to augment dynamic medial column support. To further reinforce the reconstruction and maximize inversion strength, the remaining stump of the PTT was sutured to the FDL tendon.

Step 4: Subtalar Arthroereisis. The protocol concluded with subtalar arthroereisis through the insertion of a Biopro implant (Biopro, Port Huron, MI, USA) into the sinus tarsi. This step was performed to limit excessive subtalar eversion and provide temporary hindfoot stabilization during the healing phase. The implant size and positioning were confirmed fluoroscopically to avoid overcorrection or impingement.

Postoperative management followed a standardized protocol: patients were placed in a short-leg cast for six weeks, during which immediate weight-bearing as tolerated was permitted with the assistance of a walker or crutches. Following cast removal at six weeks, a structured physical therapy program was initiated, focusing on proprioception training and progressive muscle strengthening. Patients typically resumed activities of daily living (ADLs) by two months postoperatively, and progressed to low-impact sports, such as jogging and cycling, by the third month.

Outcome measures encompassed validated tools: the Visual Analog Scale (VAS) for subjective pain, the American Orthopaedic Foot & Ankle Society (AOFAS) hindfoot score for comprehensive function (incorporating pain, activity, and alignment subscales). Outcome data were retrospectively collected through a comprehensive review of electronic medical records and imaging studies. Furthermore, arch integrity was objectively evaluated via multiple radiographic parameters, including the Meary angle (AP and lateral), calcaneal pitch, and talonavicular coverage angle. These radiographic measurements were obtained from standardized weight-bearing foot radiographs using calibrated digital tools within the institutional picture archiving and communication system (PACS). All measurements were performed according to predefined protocols, with the longitudinal axis of the talus defined by a line bisecting the head and neck of the talus to ensure standardization. Serial evaluations at predefined intervals (preoperative, 4/8/12 weeks, and 6/12 months) allow tracking of temporal changes and longitudinal evaluation of postoperative outcomes, with secondary endpoints such as implant removal rates, providing insights into complications. Data collection via a chart review is feasible in a retrospective setting. To minimize measurement bias, all radiographic images were independently evaluated by two senior orthopedic surgeons. Intra-observer and inter-observer reliability were assessed using the intraclass correlation coefficient (ICC) to ensure internal consistency and validate the precision of the radiographic assessments.

### 2.2. Statistical Analysis

To account for correlated repeated measures within individuals over time, statistical analyses were performed using generalized estimating equations (GEE) within a generalized linear model (GLM) framework. Continuous outcome variables were modeled under a Gaussian distribution with an identity link function, which was considered appropriate given the data’s distributional characteristics. An exchangeable working correlation structure was selected as the primary model to account for equal within-subject correlations across follow-up intervals, whereas sensitivity analyses using alternative correlation structures, including unstructured models, were conducted to confirm the robustness of the results.

Time was included as a categorical factor to evaluate longitudinal trends, and disease stage (stage 1a vs. 1b PCFD) was incorporated to assess baseline differences between the groups. The interaction terms between time and stage were examined to explore potential differences in outcome trajectories over the follow-up period. The multivariate models were adjusted for clinically relevant covariates, including age, sex, and body mass index, to control for potential confounding effects. The estimated marginal means with corresponding 95% confidence intervals were calculated to facilitate clinically interpretable comparisons across time points and between groups.

To evaluate the potential impact of subtalar arthroereisis on mid-term stability, a subgroup analysis was performed comparing patients who underwent implant removal with those who retained the implant. Demographics and clinical outcomes were analyzed using independent *t*-tests. Furthermore, radiographic stability was assessed across time points using interaction analysis (Time × Removal Status) within the GEE framework to determine if removal influenced the maintenance of correction.

To ensure the precision of radiographic measurements and achieve rigorous landmark identification, intra-observer and inter-observer reliability were assessed using the intraclass correlation coefficient (ICC) based on independent evaluations by two senior orthopedic surgeons. Missing data were infrequent and handled using available case analysis, as the proportion was considered insufficient to materially affect the model estimates. All statistical tests were two-sided, and significance thresholds were defined a priori at *p* < 0.05. Where appropriate, adjustments for multiple comparisons were made to maintain statistical rigor. Statistical analyses were performed using SPSS (version 27.0; IBM Corp., Armonk, NY, USA) and R (version 4.3.1; R Foundation for Statistical Computing, Vienna, Austria) to ensure transparency and reproducibility.

## 3. Results

### 3.1. Patient Demographics

Twenty patients met the inclusion criteria (19 females and 1 male). The mean age was 61.0 ± 8.3 years (range, 50 to 80 years), and the mean body mass index (BMI) was 26.9 ± 8.25 kg/m^2^ (Table 1). All patients presented with stage 1a or 1b PCFD. The mean follow-up duration was 25.8 months (range: 20–28 months). No major intra- or immediate postoperative complications have been reported to date.

### 3.2. Radiographic Outcomes

Regarding the precision of radiographic assessments, the inter-observer and intra-observer reliability showed excellent consistency. The intraclass correlation coefficients (ICCs) for the radiographic measurements were 0.91 (95% CI: 0.84–0.96) for inter-observer reliability and 0.94 (95% CI: 0.89–0.98) for intra-observer reliability, confirming the high reproducibility of the standardized landmark identification protocol.

Significant improvements were observed in both radiographic and clinical parameters following surgery (Table 2). The mean anteroposterior (AP) Meary angle decreased from 24.2° ± 7.4° preoperatively to 9.2° ± 4.1° postoperatively and was maintained at 8.6° ± 4.1° at 24 months of follow-up (*p* < 0.001). Similarly, the mean lateral Meary angle improved from 9.96° ± 2.3° preoperatively to 1.92° ± 1.4° postoperatively, with sustained correction at 2.05° ± 1.6° at 24 months (*p* < 0.01). The calcaneal pitch angle increased from 10.64° ± 4.87° preoperatively to 15.78° ± 3.33° postoperatively and remained stable at 15.55° ± 4.25° at final follow-up (*p* < 0.01). The talonavicular coverage angle showed marked improvement, decreasing from 14.53° ± 6.67° preoperatively to 3.46° ± 1.56° postoperatively, with maintenance at 3.58° ± 0.78° at 24 months (*p* < 0.024). In addition to radiographic correction, significant clinical improvements were also observed. The mean VAS pain score decreased from 7.2 ± 1.2 preoperatively to 2.6 ± 1.1 postoperatively and further improved to 1.9 ± 1.1 at final follow-up (*p* < 0.001). The AOFAS hindfoot score improved from 48.5 ± 10.3 preoperatively to 78.4 ± 8.9 postoperatively and reached 84.7 ± 7.6 at 24 months (*p* < 0.001).

### 3.3. Clinical Outcomes

Using a GEE model, VAS scores significantly improved after surgery (estimated marginal mean reduction = −4.37 points, *p* < 0.001). After adjusting for age, sex, and BMI, postoperative pain remained significantly lower than the preoperative values (*p* < 0.001).

Age and sex were also significant predictors; each additional year of age was associated with a 0.04-point decrease in VAS score (*p* = 0.03), and male patients had VAS scores 1.15 points lower than those of females. BMI was not significantly associated with changes in VAS scores (*p* > 0.05).

AOFAS hindfoot scores increased significantly from a preoperative mean of 48.5 ± 10.3 to 84.7 ± 7.6 at final follow-up (*p* = 0.001).

### 3.4. Arthroeresis Removal

Subtalar arthroereisis implant removal was performed in 9 of 20 patients (45.0%) at a mean of 5.6 ± 3.1 months postoperatively. Subgroup analysis demonstrated that preoperative baseline characteristics, including age (58.9 vs. 59.8 years), sex distribution, and side of surgery, were comparable between the Removal group (*n* = 9) and the Retention group (*n* = 11), with no statistically significant differences observed (all *p* > 0.05; Table 3). Notably, the Removal group exhibited a higher preoperative BMI (29.2 vs. 27.5 kg/m^2^), although this did not reach statistical significance (*p* = 0.125).

Despite implant removal, radiographic alignment remained stable. At final follow-up, both groups demonstrated significant improvements in radiographic parameters of Calcaneal Pitch and Talonavicular coverage angle. Interaction analysis (Time × Removal Status) showed no significant effect for any radiographic metric (*p* > 0.05), indicating similar temporal patterns of radiographic improvement in both groups (Table 4). These findings suggest that maintenance of structural correction was not dependent on continued implant retention.

Regarding clinical outcomes, no significant intergroup differences were observed in VAS or AOFAS scores at the final follow-up (*p* > 0.05; Table 4), indicating comparable long-term success in both subgroups. However, significant interaction effects (Time × Removal Status) were identified for both VAS (*p* = 0.002) and AOFAS scores (*p* = 0.041), reflecting distinct recovery trajectories (Table 4). Longitudinal analysis revealed that while both groups experienced immediate postoperative pain reduction, the Removal group demonstrated a secondary, significant improvement in VAS and AOFAS scores specifically following implant removal (Supplementary Table S1). These findings suggest that while subtalar arthroereisis provides essential early mechanical support for immediate weight-bearing, implant-related sinus tarsi pain may limit functional gains in a subset of patients. Notably, the resolution of mechanical irritation through elective removal facilitated a rapid clinical “catch-up,” ultimately allowing both groups to achieve excellent and equivalent functional outcomes.

### 3.5. Complications and Follow-Up

No wound dehiscence, infection, tendon rupture, or neurovascular injury was observed. All patients achieved full weight bearing at approximately eight weeks postoperatively and returned to daily activities without assistive devices.

### 3.6. Representative Case

**Figure 2:**
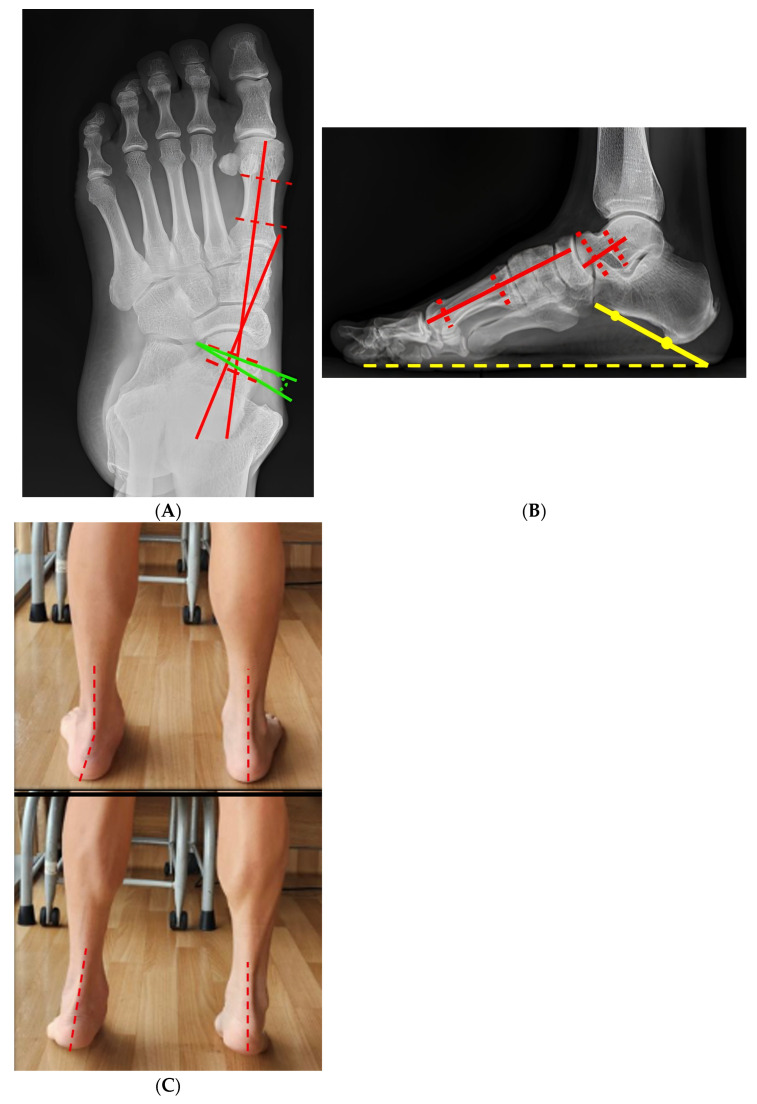
Preoperative clinical and radiographic findings. (**A**) Weight-bearing radiographs of foot AP view demonstrate marked deformity, with an anteroposterior Meary angle of 26.1° (red line) and a talonavicular coverage angle measured 15.8° (green line) (**B**) Weight-bearing radiographs of foot lateral view showed the lateral Meary angle of 9.2° (red line) and the calcaneal pitch angle was 23.8° (yellow line). (**C**) Standing clinical photographs show hindfoot valgus alignment and failure to achieve hindfoot inversion during single-leg heel rise. The red dashed line indicates the longitudinal axis of the lower leg and hindfoot.

**Figure 3:**
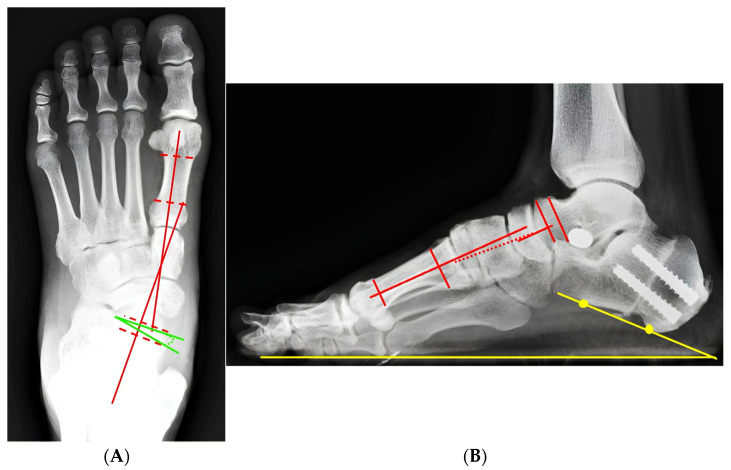
Immediate postoperative radiographic correction following standardized four-in-one reconstruction. (**A**) Weight-bearing radiographs of foot anteroposterior view demonstrate correction of alignment, with improvement of the anteroposterior Meary angle to 10.2° (red line) and the talonavicular coverage angle improved to 4.2° (green line). (**B**) The Weight-bearing radiographs of foot lateral view showed the lateral Meary angle to 4.4° (red line) and the calcaneal pitch angle was 19.7° (yellow line) with internal fixation for medializing calcaneal osteotomy and the subtalar arthroereisis implant are visible. The red dashed line indicates the longitudinal axis of the lower leg and hindfoot.

**Figure 4:**
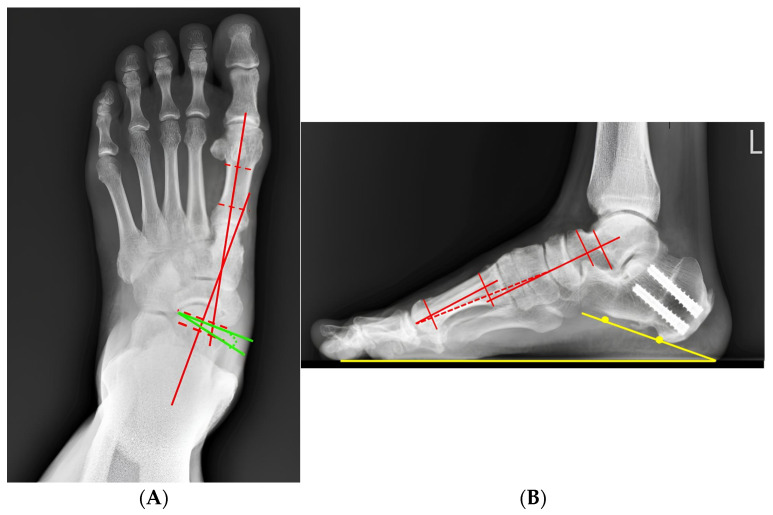
Maintained radiographic correction after elective subtalar arthroereisis removal. (**A**) Post-implant removal weight-bearing radiographs demonstrate preservation of alignment, with an anteroposterior Meary angle of 11.5° (red line) and the talonavicular coverage angle was 4.3° (green line). (**B**) The Weight-bearing radiographs of foot lateral view showed a lateral Meary angle of 4.5° (red line) and the calcaneal pitch angle was 18.9° (yellow line), without evidence of recurrent deformity or loss of correction. The red dashed line indicates the longitudinal axis of the lower leg and hindfoot.

**Figure 5:**
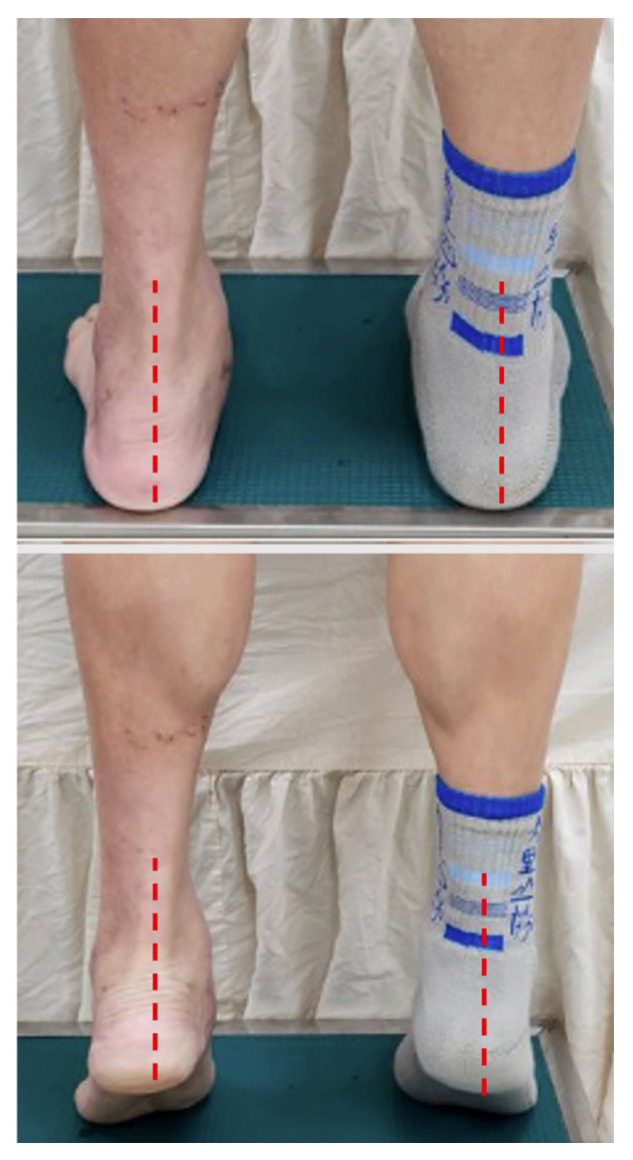
Standing hindfoot alignment photographs at three months postoperatively. The red dashed line indicates the longitudinal axis of the lower leg and hindfoot. Standing hindfoot alignment photographs demonstrate correction of valgus deformity toward a neutral alignment during standing and single-leg heel rise at three months postoperatively.

A representative patient from the cohort illustrates the typical radiographic correction achieved using the standardized four-in-one procedure. Preoperative weight-bearing radiographs demonstrated a marked deformity with an anteroposterior Meary’s angle of 26.1° and a lateral Meary’s angle of 9.2°. Postoperative radiographs showed substantial correction, which was maintained following elective removal of the subtalar arthroereisis during follow-up (Figure 2, Figure 3, Figure 4 and Figure 5).

## 4. Discussion

This study evaluated early clinical and radiographic outcomes after a standardized four-in-one reconstruction protocol for stage 1a/1b PCFD, combining MCO, FDL transfer, spring ligament repair augmented with suture-tape internal bracing, and subtalar arthroereisis [1,4,12,18]. The improvements observed in radiographic alignment, pain reduction, and functional metrics were consistent with the expected treatment effects of joint-preserving reconstruction for flexible deformities [4,11,21]. In the context of the modern PCFD nomenclature, these findings support the practical feasibility of applying a reproducible protocol-driven reconstruction sequence to address multiplanar collapses in early-stage disease [1,2].

Radiographic correction is clinically meaningful in PCFD because the deformity involves combined sagittal, coronal, and transverse plane abnormalities driven by peritalar instability and medial column failure [1,3,10]. Standardized weight-bearing imaging is recommended for assessment and follow-up. Expert consensus increasingly supports weight-bearing CT, which can refine the evaluation of peritoneal subluxation and surgical planning [9,10]. The Meary angle is widely used to quantify medial arch collapse and postoperative restoration on lateral radiographs, whereas forefoot abduction and midfoot instability are reflected in anteroposterior plane alignment metrics and peritalar relationships [3,10]. In our cohort, sustained improvements in both the anteroposterior and lateral Meary angles indicated effective restoration of the medial column alignment and hindfoot position following combined osseous and soft-tissue reconstruction [11]. Furthermore, our inclusion of calcaneal pitch and the talonavicular coverage angle provides a more comprehensive evaluation of multiplanar realignment, addressing the sagittal and transverse plane deformities that are characteristic of PCFD.

The management of PCFD has undergone a paradigm shift over the last decade, transitioning from a tendon-centric focus to comprehensive recognition of multiplanar peritalar instability. In this study, we evaluated a standardized, four-in-one reconstruction protocol designed to provide immediate mechanical stability and long-term biological healing. Our results indicate that a comprehensive, multi-component reconstruction strategy—combining osseous realignment via medializing calcaneal osteotomy (MCO), dynamic medial support through flexor digitorum longus (FDL) tendon transfer, static stabilization with spring ligament repair and internal brace augmentation, and temporary subtalar joint control using arthroereisis—produces robust radiographic correction. Notably, these clinical and radiographic improvements demonstrated high statistical significance and were maintained over time, persisting even after removal of the subtalar implant. This suggests that the standardized protocol facilitates a successful transition from initial mechanical stabilization to durable biomechanical realignment rather than implant-dependent stability.

### 4.1. The Shift from “2-in-1” to Multi-Component Reconstruction

Historically, the “2-in-1” combination of MCO and FDL transfer was the cornerstone of Stage II AAFD treatment [4,11]. Our study focuses on Stage 1a and 1b PCFD, which aligns with the contemporary nomenclature for what was previously termed Stage II AAFD. Research from the early 2010s emphasized the biomechanical effect of medial displacement calcaneal osteotomy in altering hindfoot loading mechanics and reducing valgus forces across the subtalar joint [22]. However, a growing body of evidence over the last 5–10 years suggests that the 2-in-1 approach often fails to address the “missing link” of PCFD: the incompetence of the static medial stabilizers [5,6,7]. We contend that persistent deformity and recurrence in these early-stage patients (1a/1b) are frequently related to the undertreatment of static medial restraints—particularly the spring-ligament complex and deltoid-spring restraint—rather than isolated tendon dysfunction [5,6,7].

Biomechanical work underscores that the deltoid and spring ligaments function as primary restraints against pronation and valgus collapse, and that failure of these structures can accelerate peritalar instability and arch collapse [7]. Contemporary clinical reviews emphasize the high prevalence of spring ligament pathology in PCFD and describe reconstruction strategies for restoring medial restraint [11,23]. In this context, suture-tape augmentation (“internal brace”) has been adopted to share load during early healing and to reduce the risk of elongation in soft-tissue repairs under physiologic loading [12,16,17]. Clinical series of internal brace-augmented deltoid-spring repairs have reported maintenance of correction with low early failure rates, supporting the plausibility that tape augmentation may enhance initial stability and protect biological healing [16,17].

Recent biomechanical studies have illustrated that, while FDL transfer provides dynamic support, it lacks the tensile strength to prevent long-term attenuation of the medial column if the spring ligament remains incompetent [7,12]. This has led to the emergence of “3-in-1” protocols that include spring ligament repair. However, traditional 3-in-1 repairs remain susceptible to elongation during the creeping substitution phase of ligamentous healing. Our four-in-one protocol, including suture-tape internal bracing, specifically addresses this vulnerability by acting as a load-sharing device that protects the biological repair during early physiologic loading [24]. This concept is supported by a recent clinical series reporting lower recurrence rates compared to isolated ligamentous repair [16,17]. By concurrently addressing hindfoot valgus, dynamic insufficiency, and static ligamentous failure, this comprehensive approach aims to prevent the progressive subluxation and recurrence often associated with less extensive reconstructive strategies in early-stage PCFD.

### 4.2. Comparative Analysis of Surgical Protocols

The complexity of our four-component protocol warrants comparison with simpler historical cohorts on both efficacy and morbidity (Table 5). Prior reports suggest that isolated 2-in-1 constructs (MCO + FDL) may be associated with higher rates of residual transverse-plane deformity or recurrent forefoot abduction, while 3-in-1 strategies emphasize soft-tissue reconstruction alone may be subject to gradual ligamentous attenuation over time. These observations have led to increasing interest in more comprehensive reconstruction strategies. These known limitations have motivated interest in more comprehensive, protocol-driven approaches, including the present four-component construct. Our four-in-one protocol incorporates “internal splinting” via an internal brace and temporary stabilization through arthroereisis, aiming to provide an additional layer of mechanical support during the critical early healing phase. Although this results in a higher secondary hardware removal rate (45%), this is considered a calculated trade-off for achieving the immediate and comprehensive multiplanar alignment documented in our results. While our results demonstrated improvements in multiplanar alignment, the present study design does not allow comparison with less complex or more selective reconstruction strategies. Therefore, it remains unclear whether a more comprehensive protocol provides additional benefit over alternative approaches. Further comparative, prospective studies are required to clarify the relative advantages and potential trade-offs among different surgical strategies.

### 4.3. The Paradox of Subtalar Arthroereisis in Adults

Subtalar arthroereisis remains a controversial adjunct to flexible flatfoot reconstruction in adults [18,19,20]. While it provides reliable radiographic correction, sinus tarsi pain and implant intolerance frequently lead to removal, with reported rates between 20% and 50% [18,19]. A primary clinical rationale for incorporating arthroereisis into our four-component protocol is to provide the supplemental mechanical stability necessary to facilitate immediate postoperative weight-bearing. This early mobilization is particularly beneficial for mitigating deconditioning and functional decline, especially in older populations [25]. Within this framework, the implant functions as a temporary “internal splint” that supports the foot during the critical early phase of osseous healing and biological integration of the soft-tissue reconstruction.

Previous studies suggest that reconstructions relying solely on medializing calcaneal osteotomy (MCO) and FDL transfer may be insufficient when addressing significant midfoot collapse or spring ligament incompetence, potentially leading to residual or recurrent deformity [26,27,28,29]. Our longitudinal results (Table 4) demonstrate that structural alignment remained stable despite implant removal, with both subgroups maintaining significant improvements in Calcaneal Pitch and TNCA. The non-significant interaction effect (*p* > 0.05) for radiographic outcomes further indicates that maintenance of realignment is not dependent on permanent implant retention once the underlying reconstruction has matured. These findings suggest that while arthroereisis facilitates early realignment [20,30], high-level evidence is lacking to demonstrate that routine use at the index procedure is mandatory to prevent long-term failure [18,19,31,32].

Clinical outcomes in the present cohort further elucidate the “transitional” nature of the implant. While the four-component reconstruction protocol is designed to address the multifactorial nature of PCFD—including hindfoot valgus, dynamic insufficiency, and medial ligamentous attenuation [1,4,7,12]—our data specifically highlight a distinct recovery trajectory following implant removal. Although the implant provides essential early mechanical support, it may cause symptomatic irritation in a subset of patients. Notably, our analysis revealed that elective removal resolved this irritation and facilitated a rapid functional “catch-up,” ultimately achieving excellent clinical outcomes equivalent to those in the retention group (Supplementary Table S1). In conclusion, our data suggest that subtalar arthroereisis should be regarded as an optional but valuable stabilizing adjunct rather than a permanent structural requisite. The clear benefit of enabling immediate weight-bearing must be balanced against the likelihood of a secondary procedure, and its use should be individualized based on patient-specific factors and surgeon preference.

### 4.4. Clinical Significance and Global Context

In the context of modern PCFD nomenclature, our findings support the practical feasibility of a standardized, protocol-driven approach to address multiplanar collapse. By addressing all three anatomical planes—coronal (MCO), sagittal (FDL transfer and Spring repair), and transverse (Arthroereisis and spring ligament repair)—this protocol aims to provide a predictable framework that may reduce the intraoperative decision-making complexity often associated with flexible flatfoot reconstruction.

Furthermore, the stability provided by this comprehensive stabilization strategy is particularly relevant in the aging PCFD demographic. Modern rehabilitation trends increasingly prioritize immediate weight-bearing and early mobilization to prevent sarcopenia, deconditioning, and venous thromboembolism [25]. Our protocol, reinforced by internal bracing and the adjunctive support of subtalar arthroereisis, facilitates this accelerated recovery. This strategy offers a reliable alternative to more selective reconstructions, especially when early functional restoration is a primary surgical objective. The reproducibility of these outcomes—validated by standardized measurements and high inter-observer reliability (ICC: 0.91)—establishes a consistent framework for joint-preserving surgery where the goal is anatomical, multiplanar realignment.

### 4.5. Limitations and Future Directions

Several limitations of this study should be acknowledged. First, the absence of a control group precluded us from isolating the specific incremental benefit of arthroereisis relative to that of an internal brace within the 4-in-1 reconstruction protocol. Consequently, the individual contribution of each component to overall deformity correction and clinical outcomes could not be clearly delineated. Second, while the retrospective design may introduce inherent biases, we addressed potential measurement variability by implementing a standardized landmark identification protocol, which was validated by high inter-observer and intra-observer reliability (ICC: 0.91–0.94). Third, the marked gender imbalance in our cohort (19 females and 1 male) may limit the generalizability of our findings. Given the sex-related differences in ligamentous laxity and biomechanical characteristics [3,30], this predominance of female patients may have influenced both deformity progression and the postoperative response. Furthermore, while we have established the stability of radiographic correction following implant removal, longer-term follow-up is essential to monitor for late recurrence or progressive functional changes [31,33,34]. Future prospective, comparative studies are required to clarify whether the routine use of arthroereisis provides a definitive advantage over more selective reconstructive approaches. Specifically, further investigation should evaluate if the benefits of immediate weight-bearing and early mobilization sufficiently justify the relatively high rate of secondary implant removal.

## 5. Conclusions

The four-in-one reconstruction protocol—integrating medializing calcaneal osteotomy, spring ligament repair, flexor digitorum longus (FDL) tendon transfer with internal brace augmentation, and subtalar arthroereisis—offers a comprehensive, multiplanar approach for stage 1a/1b PCFD. Our findings demonstrate that this standardized strategy provides significant and stable clinical and radiographic improvements within a retrospective cohort. Notably, while the 45% implant removal rate highlights the high incidence of sinus tarsi intolerance, radiographic alignment was successfully maintained following explantation. This suggests that the combined effect of osseous realignment and ligamentous reconstruction provides sufficient structural integrity once biological healing is complete, rendering the arthroereisis implant a functional but temporary adjunct.

Crucially, the primary clinical value of including arthroereisis in this protocol lies in its role as an “internal splint” to facilitate immediate postoperative weight-bearing and early mobilization. However, the lack of a comparative control group precludes definitive conclusions regarding its independent necessity or superiority over selective, “à la carte” surgical approaches. Future prospective, controlled studies are essential to delineate the incremental contributions of temporary arthroereisis and internal bracing, ultimately defining the most cost-effective and evidence-based strategy for progressive flatfoot reconstruction.

## Figures and Tables

**Table 1:** Patient demographics and baseline characteristics.

Variable	Value
Total patients (*n*)	20
Female:Male	19:1
Mean age (years)	61.0 ± 8.3
BMI (kg/m^2^)	26.9 ± 8.25
Affected side (Right:Left)	8:12

**Table 2:** Radiographic and clinical outcomes before and after surgery.

Parameter	Preoperative	Postoperative	24 Months of Follow-Up	*p*-Value
Meary angle (AP, °)	24.2 ± 7.4	9.2 ± 4.1	8.6 ± 4.1	<0.001
Meary angle (Lateral, °)	9.96 ± 2.3	1.92 ± 1.4	2.05 ± 1.6	<0.012
Calcaneal pitch (°)	10.64 ± 4.87	15.78 ± 3.33	15.55 ± 4.25	<0.01
Talonavicular coverage angle (°)	14.53 ± 6.67	3.46 ± 1.56	3.58 ± 0.78	<0.024
VAS pain score	7.2 ± 1.2	2.6 ± 1.1	1.9 ± 1.1	<0.001
AOFAS hindfoot score	48.5 ± 10.3	78.4 ± 8.9	84.7 ± 7.6	<0.001

**Table 3:** Demographics Comparison between Groups.

Variable	Removed (*n* = 9)	Retention (*n* = 11)	*p*-Value
Age (years)	60.1 ± 7.9	60.9 ± 8.6	0.833
BMI (kg/m^2^)	27.8 ± 3.5	26.1 ± 4.1	0.332
Side (Right)	5 (55.6%)	6 (54.5%)	0.999

**Table 4:** Radiographic and clinical Outcomes Comparison between Groups.

Radiographic Parameter	Group	Preoperative	Final Follow-Up	*p*-Value (Final)	*p*-Value (Interaction)
Meary angle (AP, °)	Removed	24.2 ± 7.4	8.6 ± 4.1	0.912	0.884
	Retention	23.8 ± 6.9	8.8 ± 3.9		
Meary angle (Lat, °)	Removed	10.9 ± 2.1	2.0 ± 0.3	0.724	0.920
	Retention	8.8 ± 1.9	2.0 ± 0.5		
Calcaneal Pitch (°)	Removed	12.9 ± 2.3	15.5 ± 2.1	<0.01 *	0.752
	Retention	12.4 ± 6.3	16.2 ± 4.3		
Talonavicular coverage angle (°)	Removed	11.0 ± 2.2	3.8 ± 1.8	0.024 *	0.814
	Retention	17.1 ± 7.9	3.3 ± 1.5		
**Clinical Outcome**	**Group**	**Preoperative**	**Final Follow-Up**	***p*-Value (Final)**	***p*-Value (Interaction)**
VAS Score	Removed	7.8 ± 0.4	2.0 ± 0.7	0.57	0.002 *
	Retention	6.7 ± 0.8	2.2 ± 0.8		
AOFAS Score	Removed	52.2 ± 5.7	91.1 ± 3.3	0.125	0.041 *
	Retention	57.5 ± 6.3	88.1 ± 4.8		

Data are presented as mean ± standard deviation. *p*-values (Final) represent between-group comparisons at final follow-up. *p*-values (Interaction) represent time × group interaction effects. * *p* < 0.05 was considered statistically significant.

**Table 5:** Comparative Efficacy and Morbidity of Multimodal vs. Traditional Protocols.

Protocol	Components	10-Year Literature Trend	Clinical Utility vs. Risks
2-in-1	MCO + FDL	Risk of residual transverse-plane deformity in selected patterns [4,5].	Lower surgical time; higher risk of recurrent midfoot abduction.
3-in-1	MCO + FDL + SL Repair	Recognition of the Spring Ligament (SL) as the primary stabilizer [11].	Improved arch height; potential for soft-tissue stretching over time.
4-in-1 (Current)	MCO + FDL + SL/IB + Arthroereisis	Focus on “Internal Splinting” and immediate stabilization [12,18].	Protocolized multiplanar correction; high hardware removal rate (45%).

## Data Availability

Anonymized data not published within this article will be made available by request from any qualified investigator.

## Supplementary Materials

The following supporting information can be downloaded at: https://www.mdpi.com/article/10.3390/diagnostics16081124/s1, Table S1. Comparison of Longitudinal Clinical Outcomes Between Patients with Subtalar Arthroereisis Removal and Retention.

## Abbreviations

The following abbreviations are used in this manuscript:

AAFD	adult-acquired flatfoot deformity
AOFAS	American Orthopaedic Foot & Ankle Society
BMI	body mass index
FDL	flexor digitorum longus
GEE	generalized estimating equations
GLM	generalized linear model
IRB	institutional review board
MCO	medializing calcaneal osteotomy
PCFD	progressive collapsing foot deformity
SL	spring ligament
VAS	visual analog scale

## References

[1] Myerson M.S., Thordarson D.B., Johnson J.E., Hintermann B., Sangeorzan B.J., Deland J.T., Schon L.C., Ellis S.J., de Cesar Netto C. (2020). Progressive collapsing foot deformity. Foot Ankle Int..

[2] Pasapula C.S., Choudkhuri M.R., Monzó E.R.G., Dhukaram V., Shariff S., Pasterse V., Richie D., Kobezda T., Solomou G., Cutts S. (2024). Review of classification systems for adult acquired flatfoot deformity/progressive collapsing foot deformity and development of a triple classification. J. Clin. Med..

[3] Flores D.V., Mejía Gómez C., Fernández Hernando M., Davis M.A., Pathria M.N. (2019). Adult acquired flatfoot deformity: Anatomy, biomechanics, staging, and imaging findings. Radiographics.

[4] Henry J.K., Shakked R., Ellis S.J. (2019). Adult-acquired flatfoot deformity. Foot Ankle Orthop..

[5] Krautmann K., Kadakia A.R. (2021). Spring and deltoid ligament insufficiency in progressive collapsing foot deformity. Foot Ankle Clin..

[6] Jackson J.B. (2022). Adult acquired flatfoot deformity. J. Am. Acad. Orthop. Surg..

[7] Hintermann B. (2021). Biomechanics of medial ankle and peritalar instability. Foot Ankle Clin..

[8] de Cesar Netto C., Silva T., Li S., Mansur N.S., Auch E., Dibbern K., Femino J.E., Baumfeld D. (2020). Assessment of Posterior and Middle Facet Subluxation of the Subtalar Joint in Progressive Flatfoot Deformity. Foot Ankle Int..

[9] de Cesar Netto C., Myerson M.S., Day J., Ellis S.J., Hintermann B., Johnson J.E., Sangeorzan B.J., Schon L.C., Thordarson D.B., Deland J.T. (2020). Consensus for the Use of Weightbearing CT in the Assessment of Progressive Collapsing Foot Deformity. Foot Ankle Int..

[10] Polichetti C., De Filippo M., Sverzellati N. (2023). Imaging of adult acquired flatfoot deformity. Diagnostics.

[11] Chien B.Y., Greisberg J.K., Arciero E. (2023). Spring ligament reconstruction for progressive collapsing foot deformity: Contemporary review. Foot Ankle Int..

[12] Nery C., Lemos A.V.K.C., Raduan F., Mansur N.S.B., Baumfeld D. (2018). Combined spring and deltoid ligament repair in adult-acquired flatfoot deformity. Foot Ankle Int..

[13] Brodell J.D., MacDonald A., Perkins J.A., Deland J.T., Oh I. (2019). Deltoid-Spring Ligament Reconstruction in Adult Acquired Flatfoot Deformity With Medial Peritalar Instability. Foot Ankle Int..

[14] Osbeck I., Cöster M., Montgomery F., Atroshi I. (2023). Surgically treated adult acquired flatfoot deformity: Register-based study. Foot Ankle Surg..

[15] Conti M.S., Jones M.T., Savenkov O., Deland J.T., Ellis S.J. (2018). Outcomes of reconstruction of stage II adult-acquired flatfoot deformity in older patients. Foot Ankle Int..

[16] Bastias G.F., Dalmau-Pastor M., Astudillo C., Pellegrini M.J. (2018). Spring ligament instability. Foot Ankle Clin..

[17] Tang C.Y.K., Ng K.H. (2020). Clinical and radiological outcomes of braided suture tape system augmentation for spring ligament repair in flexible flatfoot. Foot.

[18] Mattesi L., Ancelin D., Severyns M.P. (2021). Is subtalar arthroereisis a good procedure in adult acquired flatfoot? A systematic review. Orthop. Traumatol. Surg. Res..

[19] Baryeh K.W., Ismail H., Sobti A., Harb Z. (2022). Outcomes following subtalar arthroereisis in adult acquired flatfoot. Foot Ankle Spec..

[20] Saxena A., Via A.G., Maffulli N., Chiu H. (2016). Subtalar arthroereisis implant removal in adults. J. Foot Ankle Surg..

[21] Mueller G., Frosch K.-H., Barg A., Schlickewei C., Weel H., Krähenbühl N., Priemel M. (2024). Impact of medial displacement calcaneal osteotomy on foot biomechanics. Arch. Orthop. Trauma Surg..

[22] Arangio G.A., Salathe E.P. (2009). A biomechanical analysis of posterior tibial tendon dysfunction, medial displacement calcaneal osteotomy and flexor digitorum longus transfer in adult acquired flat foot. Clin. Biomech..

[23] Krautmann K., Kadakia A.R. (2021). Spring and deltoid ligament insufficiency in the setting of progressive collapsing foot deformity: An update on diagnosis and management. Foot Ankle Clin..

[24] Marsland D., Morris A.M., Gould A.E.R., Calder J.D.F., Amis A.A. (2022). Systematic review of tendon transfers in the foot and ankle using interference screw fixation: Outcomes and safety of early versus standard postoperative rehabilitation. Foot Ankle Surg..

[25] Cruz-Jentoft A.J., Bahat G., Bauer J., Boirie Y., Bruyère O., Cederholm T., Cooper C., Landi F., Rolland Y., Sayer A.A. (2019). Sarcopenia: Revised European consensus on definition and diagnosis. Age Ageing.

[26] Hintermann B., Knupp M., Pagenstert G. (2007). Surgical treatment of the adult acquired flexible flatfoot. Foot Ankle Clin..

[27] Niki H., Hirano T., Okada H., Beppu M. (2012). Outcome of medial displacement calcaneal osteotomy for correction of adult-acquired flatfoot. Foot Ankle Int..

[28] Hintermann B., Valderrabano V., Nigg B.M. (2006). Correction of moderate and severe acquired flexible flatfoot with medializing calcaneal osteotomy and flexor digitorum longus transfer. J. Bone Joint Surg. Am..

[29] Maskill J.D., Bohay D.R., Anderson J.G. (2010). Radiographic correction following reconstruction of adult acquired flatfoot deformity. Foot Ankle Int..

[30] Abousayed M.M., Alley M.C., Shakked R., Rosenbaum A.J. (2017). Adult-acquired flatfoot deformity: Etiology, diagnosis, and management. JBJS Rev..

[31] De Pellegrin M., Moharamzadeh D. (2021). Subtalar arthroereisis for surgical treatment of flexible flatfoot. Foot Ankle Clin..

[32] Walley K.C., Greene G., Hallam J., Juliano P.J., Aynardi M.C. (2019). Short- to mid-term outcomes following the use of an arthroereisis implant as an adjunct for correction of flexible, acquired flatfoot deformity in adults. Foot Ankle Spec..

[33] de Cesar Netto C., Saito G.H., Roney A., Day J., Greditzer H., Sofka C., Ellis S.J., Richter M., Barg A., Lintz F. (2021). Combined weightbearing CT and MRI assessment of flexible progressive collapsing foot deformity. Foot Ankle Surg..

[34] Poutoglidou F., Marsland D., Elliot R. (2024). Does foot shape really matter? Correlation of patient-reported outcomes with radiographic assessment in progressive collapsing foot deformity reconstruction: A systematic review. Foot Ankle Surg..

