# Supplementary material for: Multimodal Surgical Management of Stage 1a/1b PCFD (Stage II AAFD): Early Outcomes of a Standardized Four-in-One Procedure Protocol
Source: Diagnostics (Basel). 2026 Apr 9;16(8):1124. doi: 10.3390/diagnostics16081124 (PMC13114831; doi:10.3390/diagnostics16081124)
Supplement: Supplementary file 1 [file diagnostics-16-01124-s001.zip › diagnostics-4201518-supplementary.pdf]

Supplementary Tables

Supplementary table S1. Comparison of Longitudinal Clinical Outcomes Between Patients with Subtalar Arthroereisis Removal and Retention.

Parameter	Time point	Removal (n=0)	Retention (n=11)	Between-group p-value
VAS (0-10)	Preoperative	7.0 (6.0-8.0)	5.0 (4.0-6.0)	0.015*
	4 weeks	4.0 (3.0-5.0)	4.0 (3.0-5.0)	0.817
	12 Weeks (Pre-removal)	4.0 (3.0-5.0)	3.0 (2.0-4.0)	0.180
	6 Months (Post-removal)	1.0 (1.0-2.0)	3.0 (2.0-4.0)	0.001*
	Final	1.0 (1.0-2.0)	2.5 (2.0-3.5)	0.001*
AOFAS (0-100)	Preoperative	50.0 (42.0-58.0)	55.0 (48.0-62.0)	0.285
	4 weeks	65.0 (58.0-72.0)	62.0 (55.0-69.0)	0.62
	12 Weeks (Pre-removal)	68.0 (60.0-75.0)	70.0 (63.0-77.0)	0.865
	6 Months (Post-removal)	90.0 (85.0-95.0)	85.0 (78.0-92.0)	0.038*
	Final	91.0 (86.0-96.0)	88.0 (81.0-94.0)	0.125
